# Supplementary material for: The Mental Health Impact of the COVID-19 Pandemic on Older Adults in China: A Systematic Review
Source: Int J Environ Res Public Health. 2022 Nov 2;19(21):14362. doi: 10.3390/ijerph192114362 (PMC9657377; doi:10.3390/ijerph192114362)
Supplement: Supplementary file 1 [file ijerph-19-14362-s001.zip › ijerph-1968683-supplementary.pdf]

International Journal of
Environmental Research and Public Health

MDPI

Supplementary Materials

Table S1. JBI Checklist for Assessing Risk of Bias.

Study	Were the Criteria for Inclusion in the Sample Clearly Defined?	Were the Study Subjects and the Setting Described in Detail?	Was the Exposure Measured in a Valid and Reliable Way?	Were Objective, Standard Criteria Used for Measurement of the Condition?	Were Confounding Factors Identified?	Were Strategies to Deal with Confounding Factors Stated?	Were the Outcomes Measured in a Valid and Reliable Way?	Was Appropriate Statistical Analysis Used?	Overall Appraisal:
**Cross-Sectional Studies**									
Zhou, Chen, et al.	+	+	N/A	N/A	+	+	+	+	High
Liang, et al.	+	+	N/A	N/A	+	+	+	+	High
Chen, Chen & Zhong	+	+	N/A	N/A	+	+	+	+	High
Jiang et al., 2021	+	+	N/A	N/A	+	+	-	+	Moderate
Liang & Deng., 2022	-	+	N/A	N/A	+	+	-	+	Moderate
Sun et al., 2020	+	+	N/A	N/A	+	+	+	+	High
Lu et al., 2021	-	+	N/A	N/A	+	+	+	+	Moderate
Wang, 2021	+	+	N/A	N/A	+	+	-	+	Moderate
Tao et al., 2021	+	-	N/A	N/A	+	+	+	+	Moderate
Liu & Liu, 2021	-	+	N/A	N/A	+	-	-	+	Low
Bao et al., 2021	+	+	N/A	N/A	+	+	+	+	High
Ye & Lin, 2021	+	+	N/A	N/A	+	+	+	+	High
Wang et al., 2021	+	+	N/A	N/A	+	+	+	+	High
Xu, 2022	+	+	N/A	+	+	+	+	+	High
Wang et al., 2021	+	+	N/A	+	+	+	+	+	High
Qi et al., 2021	+	+	N/A	+	+	+	+	+	High

Xue et al., 2021	+	+	N/A	+	+	+	+	+	High
Su et al., 2021	-	+	N/A	+	+	+	+	+	Moderate

Cohort Studies

Study	Were the two Groups Similar and Recruited from the Same Population?	Were the Exposures Measured Similarly to Assign People to both Exposed and Unexposed Groups?	Was the Exposure Measured in a Valid and Reliable Way?	Were Confounding Factors Identified?	Were Strategies to Deal with Confounding Factors Stated?	Were the Groups/Participants Free of the Outcome at the Start of the Study (or at the Moment of Exposure)?	Were the Outcomes Measured in a Valid and Reliable Way?	Was the Follow up Time Reported and Sufficient to be Long Enough for Outcomes to Occur?	Was Follow up Complete, and if not, Were the Reasons to Loss to Follow up Described and Explored?	Were Strategies to Address Incomplete Follow up Utilized	Was Appropriate Statistical Analysis Used?	Overall Appraisal:
Li et al.	N/A	N/A	N/A	+	+	N/A	+	+	+	N/A	+	High

Table S2. MMAT Checklist for Assessing Risk of Bias.

Category of Study Designs	Methodological Quality Criteria	Yes	No	Can't Tell	Comments
Screening questions (for all types)	S1. Are there clear research questions?	+			Moderate
	S2. Do the collected data allow to address the research questions?	+			
	Further appraisal may not be feasible or appropriate when the answer is 'No' or 'Can't tell' to one or both screening questions				
1. Qualitative	1.1 Is the qualitative approach appropriate to answer the research question?	+			
	1.2 Are the qualitative data collection methods adequate to address the research question?	+			
	1.3 Are the findings adequately derived from the data?			+	
	1.4 Is the interpretation of results sufficiently substantiated by data?			+	
	1.5 is there coherence between qualitative data sources, collection, analysis and interpretation?			+	
2. Quantitative randomized controlled trails	2.1 Is randomization appropriately performed?				
	2.2 Are the groups comparable at baseline?				
	2.3 Are there complete outcome data?				
	2.4 Are outcome assessors blinded to the intervention provided?				
	2.5 Did the participants adhere to the assigned intervention?				
3. Quantitative nonrandomized	3.1 Are the participants representative of the target population?		-		
	3.2 Are measurements appropriate regarding both the outcome and intervention (or exposure)?	+			
	3.3 Are there complete outcome data?	+			
	3.4 Are the confounders accounted for in the design and analysis?	+			
	3.5 During the study period, is the intervention administered (or exposure occurred) as intended?	+			
4. Quantitative descriptive	4.1 Is the sampling strategy relevant to address the research question?				
	4.2 is the sample representative of the target population?				
	4.3 Are the measurements appropriate?				
	4.4 Is the risk of nonresponse bias low?				
	4.5 Is the statistical analysis appropriate to answer the research question?				

5.	Mixed methods	5.1 Is there an adequate rationale for using a mixed methods design to address the research question?		+	
		5.2 Are the different components of the study effectively integrated to answer the research question?		+	
		5.3 Are the outputs of the integration of qualitative and quantitative components adequately interpreted?		+	
		5.4 Are divergences and inconsistencies between quantitative and qualitative results adequately addressed?		+	
		5.5 Do the different components of the study adhere to the quality criteria of each tradition of the methods involved?			+
